# Effects of a high-protein, low-glycemic-index diet on body weight and waist circumference in late postmenopausal women: a randomized controlled trial

**DOI:** 10.20945/2359-4292-2023-0370

**Published:** 2024-08-26

**Authors:** Thaís Rasia Silva, Suzana Cardona Lago, Tayane Muniz Fighera, Poli Mara Spritzer

**Affiliations:** 1 Universidade Federal do Rio Grande do Sul Programa de Pós-graduação em Endocrinologia e Metabolismo Porto Alegre RS Brasil Programa de Pós-graduação em Endocrinologia e Metabolismo, Universidade Federal do Rio Grande do Sul, Porto Alegre, RS, Brasil; 2 Hospital de Clínicas de Porto Alegre Serviço de Endocrinologia Unidade de Endocrinologia Ginecológica Porto Alegre RS Brasil Unidade de Endocrinologia Ginecológica, Serviço de Endocrinologia, Hospital de Clínicas de Porto Alegre (HCPA), Porto Alegre, RS, Brasil; 3 Universidade Federal do Rio Grande do Sul Laboratório de Endocrinologia Molecular Departamento de Fisiologia Porto Alegre RS Brasil Departamento de Fisiologia, Laboratório de Endocrinologia Molecular, Universidade Federal do Rio Grande do Sul (UFRGS), Porto Alegre, RS, Brasil

**Keywords:** High-protein diet, low-glycemic-index diet, postmenopause, aged, weight loss, waist circumferece

## Abstract

**Objective:**

To describe the secondary outcomes (weight loss, waist circumference [WC], and glycolipid profile) of a previous randomized controlled trial designed to investigate the impact of a high-protein, low-glycemic-index (GI) diet on lean body mass in late postmenopausal women.

**Subjects and methods:**

A total of 26 healthy women aged ≥ 65 years and with a mean body mass index (BMI) of 26.1 ± 3.5 kg/m^2^ were randomly assigned to follow a low-GI diet (GI < 55) with protein consumption at the current recommended dietary allowance (RDA, 0.8 g/kg body weight) or twice the RDA (2RDA, 1.6 g/kg body weight). Changes in body weight, BMI, WC, glucose, homeostasis model assessment of insulin resistance (HOMA-IR), total cholesterol, and triglycerides were assessed at 3- and 6-month of follow-up in all participants. An intention-to-treat analysis was performed using a linear mixed model.

**Results:**

Weight loss (mean change -1.7 kg, 95% confidence interval [CI] -2.8 to -0.5 kg, p = 0.004) was observed at 6 months, with no significant difference between the RDA and 2RDA groups. An overall significant WC decrease was observed at 6 months in all participants (mean change -3.8 cm, 95% CI -5.5 to -2.1 cm, p < 0.001), with no differences between groups. The glycolipid profile remained unchanged after 6 months of dietary intervention.

**Conclusion:**

Increasing protein intake did not lead to greater weight loss or reduction in WC in a sample of healthy postmenopausal women following a low-GI diet for 6 months.

## INTRODUCTION

Sex hormones can regulate metabolic flexibility, which determines how nutrients are converted into energy (1). With estrogen reduction during the menopausal transition, there is a tendency toward weight gain accompanied by increased abdominal fat distribution that continues into postmenopause (2,3). Furthermore, increased abdominal fat has been associated with increased cardiovascular risk even in postmenopausal women with normal body mass index (BMI) (4,5).

Regarding weight loss, no single diet has a clear superiority over others (3). However, a systematic review including data from six randomized controlled trials (RCTs) reported a significantly greater decrease in total fat mass in participants receiving a low-glycemic-index (GI) diet than in those receiving a control diet (6). Moreover, the role of GI and protein intake as cardiovascular risk markers has been investigated in healthy individuals with overweight or obesity on a non-energy-restricted diet, and the low-GI diet appears to be the most effective in maintaining weight loss, especially with moderate protein intake (7),and in reducing cardiovascular risk (8). Indeed, a scientific consensus has highlighted the importance of emerging evidence supporting low-GI diets, especially with high protein intake, in reducing total body fat mass, managing weight, and reducing the risk of cardiovascular disease (9). A previous cross-sectional study in postmenopausal women from Southern Brazil has demonstrated that adherence to a dietary pattern rich in foods with a high GI was associated with higher BMI and WC (10). However, it is important to note that the overall effectiveness of low-GI diets for weight control in postmenopausal women remains unclear.

We recently reported the clinical results of a RCT in healthy postmenopausal women, which demonstrated that a low-GI diet within the current recommended dietary allowance (RDA) of protein (0.8 g/kg body weight) and aimed at maintaining energy balance was sufficient for maintaining lean body mass (LBM) and physical performance (11). In the present study, we performed secondary analyses of the same RCT to evaluate other exploratory outcomes of the 6-month dietary intervention, namely weight loss, waist circumference (WC), and glycolipid profile.

## SUBJECTS AND METHODS

### Participants and study design

This parallel-group RCT, registered at ClinicalTrials.gov as NCT03652584, investigated the effects of either high- or moderate-protein, low-GI diet on LBM, muscle strength, and physical performance, as previously described (11). Briefly, the RCT included data from 26 apparently healthy postmenopausal women aged ≥ 65 years without systemic hormone therapy in the previous 3 months, who had been evaluated at baseline and after 3 and 6 months of dietary intervention between 2017 and 2018, in the *Hospital de Clínicas de Porto Alegre* (HCPA) research facility. Written informed consent was obtained from all participants. The study protocol was approved by the ethics committee of HCPA (CAAE: 61278916.7.0000.5327), and the study was conducted in accordance with the Helsinki Declaration. This is a secondary analysis of the previously published primary analysis, further details including exclusion criteria and sample size calculations can be found elsewhere (11). The data from this secondary analysis – weight, WC, and glycolipid profile – are reported for the first time in the present study.

### Dietary intervention

The design of the two diets of this RCT was previously reported in detail (11). In short, two low-GI diets (GI < 55) with controlled protein consumption were used: (A) at the current RDA for protein (0.8 g/kg body weight) or (B) at twice the current RDA (2RDA) for protein (1.6 g/kg body weight). Daily energy requirements were calculated and individually set for each participant to match their estimated energy expenditure, obtained by indirect calorimetry (Fitmate, Cosmed, Rome, Italy). The GI of each diet was estimated as proposed by the Food and Agriculture Organization (12). The GI of each food was extracted from the International Table of Glycemic Index, using glucose as the reference food (13). The RDA diet consisted of 15% protein, 35% fat, and 50% carbohydrate, while the 2RDA diet consisted of 30% protein, 25% fat, and 45% carbohydrate. Participants with 25-hydroxyvitamin D levels below 30 ng/mL were supplemented with vitamin D3 7,000 IU per week. To assess dietary compliance, 24-hour urinary nitrogen excretion was measured during the study as a crude marker of protein intake (14). A dietician (T.R.S.) collected information about dietary intake through face-to-face interviews at baseline and at 3 and 6 months using a validated food frequency questionnaire (FFQ). Additionally, adherence to the prescribed diets was evaluated by monthly follow-up 3-day diet food recalls. The meals were self-prepared by the participants at home.

### Outcomes

Anthropometric measurements were performed in duplicate and included body weight and WC measured with the participant standing upright. The WC was measured at the midpoint between the lowest rib and the iliac crest, perpendicular to the long axis of the body.

Blood samples were collected after a 12-hour fast at baseline and at 3- and 6-month of follow-up. Total cholesterol, high-density lipoprotein cholesterol (HDL-c), and triglyceride levels were determined using a colorimetric enzymatic method (Bayer 1800 Advia System, Deerfield, IL, USA), with intra-assay and interassay coefficients of variation (CVs) < 3%. Low-density lipoprotein cholesterol (LDL-c) was determined indirectly by the Friedewald formula: LDL-c=total cholesterol minus(HDL-c minus triglycerides/5) (15). Glucose was determined by the hexokinase method (Advia 1800) with intra-assay CV < 3.4% and interassay CV < 2.1%. Plasma insulin levels were measured using electrochemiluminescence immunoassay (Centaur XP, Siemens). Insulin resistance was estimated by the homeostasis model assessment of insulin resistance (HOMA-IR), calculated as follows: insulin(mU/L)×glucose(mmol/L)/22.5 (16).

### Statistical analysis

The results are presented as mean (standard error [SE]). Outcome data were analyzed by intention-to-treat using a mixed-effects regression model to compare diets and assessments (mid-treatment *versus* end-of-treatment). *Post hoc* comparisons were conducted using Bonferroni correction. Mean post-randomization changes in the outcomes were calculated to assess the magnitude of differences over time, considering the entire sample, and expressed with 95% confidence intervals (CIs) and SEs. Mean between-group differences were estimated using treatment contrasts and 95% CIs. The software SPSS, version 21 for Windows (IBM, Armonk, NY, USA) was used for statistical analysis. A p value < 0.05 was considered statistically significant.

## RESULTS

Table 1 shows the values for BMI, WC, and glycolipid profile at baseline and after 3 and 6 months of dietary intervention. At baseline, the 2RDA group had lower mean BMI and WC than the RDA group. The glycolipid profile was within the reference range for healthy older individuals and did not differ significantly between the groups.

**Table 1:** Body mass index, waist circumference, and glycolipid profile at baseline and after 3 and 6 months of dietary intervention (n = 26)

Variable	2RDA			RDA			Effect^a^		
	Baseline	3-month follow-up	6-month follow-up	Baseline	3-month follow-up	6-month follow-up	Diet	Time	Time x diet
BMI, kg/m²	24.5 ± 0.9^b^	24.0 ± 0.8^b^^c^	23.8 ± 0.9^b^,^c^	27.8 ± 0.9	27.3 ± 0.8^c^	27.1 ± 0.9^c^	0.011	0.006	0.979
Waist circumference, cm	77.7 ± 2.1^b^	75.6 ± 2.1^c^	74.3 ± 2.1^c^	84.7 ± 2.1	81.4 ± 2.1^c^	80.5 ± 2.1^c^	0.041	<0.001	0.490
Glucose, mg/dL	86.5 ± 5.4	88.5 ± 5.0	88.5 ± 5.0	83.4 ± 5.4	82.7 ± 5.0	83.7 ± 5.0	0.531	0.668	0.576
HOMA-IR	1.3 ± 0.2	1.3 ± 0.2	1.2 ± 0.2	1.3 ± 0.2	1.4 ± 0.2	1.5 ± 0.2	0.598	0.662	0.153
Total cholesterol, mg/dL	200.9 ± 12.6	194.8 ± 13.1	190.8 ± 12.4	191.2 ± 12.6	191.4 ± 13.1	189.7 ± 12.5	0.786	0.538	0.692
HDL cholesterol, mg/dL	63.2 ± 5.6	62.4 ± 5.7	61.1 ± 6.0	74.4 ± 5.6	71.0 ± 5.7	71.4 ± 6.1	0.204	0.221	0.639
LDL cholesterol, mg/dL	113.5 ± 10.8	111.2 ± 10.6	109.8 ± 10.4	98.8 ± 10.8	101.6 ± 10.6	100.1 ± 10.5	0.428	0.948	0.775
Triglycerides, mg/dL	119.4 ± 12.1	106.1 ± 12.2	99.4 ± 10.4	90.4 ± 12.1	94.1 ± 12.2	90.4 ± 10.5	0.275	0.370	0.302

Values are shown as mean ± standard error.

Abbreviations: BMI, body mass index; HDL, high-density lipoprotein; HOMA-IR, homeostasis model of assessment of insulin resistance; LDL, low-density lipoprotein; RDA, recommended dietary allowance, 0.8 g protein/kg body weight; 2RDA, 1.6 g/kg body weight.

a Estimates are derived from a mixed-effects regression model used to compare diets and mid-treatment and end-of-treatment assessments.

b Different between diets at indicated time point, p < 0.005.

c Different from pre-intervention within the same group, p < 0.005; the p values were determined using Bonferroni *post hoc* test.

**Figure 1:**
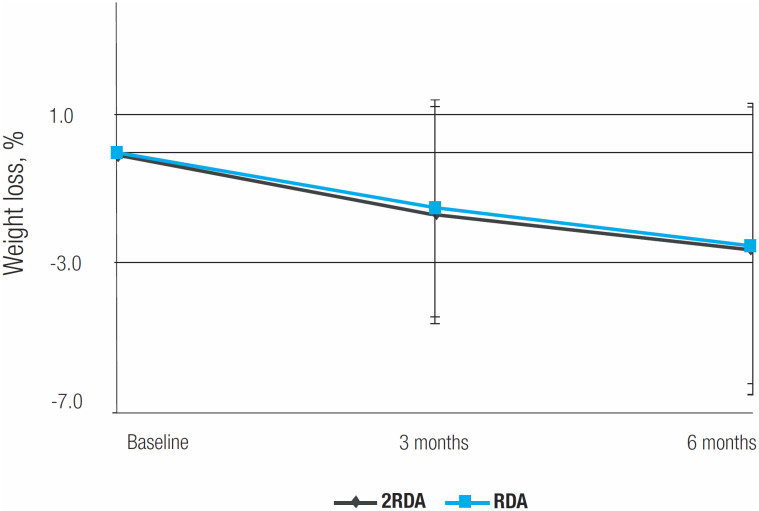
Percentage change in body weight assessed at baseline (0 months) and after 3 and 6 months of intervention. *P < 0.05 for difference over time considering the entire sample derived from the mixed-effects regression model, magnitude of change. Error bars indicate standard errors.

Abbreviations: RDA, recommended dietary allowance, 0.8 g protein/kg body weight; 2RDA, 1.6 g/kg body weight.

During intervention, BMI and WC decreased significantly regardless of the level of protein intake. In fact, the low-GI diet led to weight loss (mean change -1.7 kg, 95% CI -2.8 to -0.5 kg, p = 0.004, mixed-effects regression model) with no significant difference in the magnitude of change between the groups (Figure 1). The mean change in WC was -3.8 cm (95% CI -5.5 to -2.1 cm, p < 0.001, mixed-effects regression model), with no significant effect of the level of protein intake on WC over 6 months of dietary intervention. These results were independent of routine physical activity, which remained unchanged throughout the study period, as previously reported (11). Glucose, total cholesterol, HDL-c, LDL-c, and triglyceride levels remained unchanged after the low-GI dietary intervention (Table 1).

## DISCUSSION

In the present study, we observed that protein intake exceeding the RDA, compared with intake within the recommended RDA, did not lead to greater weight loss or reduction in WC in a sample of healthy postmenopausal women following a low-GI diet for 6 months. Importantly, women had a healthy glycolipid profile at baseline, which remained unchanged over 6 months of dietary intervention. To our knowledge, no published study has conducted a long-term evaluation of the effects of a low-GI diet on body weight and glycolipid profile in late postmenopausal women.

The primary finding of this study was that the participants lost weight despite following a dietary intervention aimed at balancing energy needs and maintaining an unchanged total caloric intake during the study period. Additionally, no significant difference in the mean percentage of weight loss was observed between the RDA and 2RDA groups. While high-protein intake can be effective for weight loss due to its increased effect on thermogenesis and LBM, we did not observe a significant change in these parameters between postmenopausal women who consumed twice the RDA for protein intake compared with those who followed the current RDA for protein intake in this 6-month follow-up trial. In this sense, we hypothesized that it was the low-GI diet that was responsible for the weight loss, which could be explained by a greater impact on stool energy excretion, consistent with previous evidence in postmenopausal women (17). Given that the role of low-GI diets in body weight management is mainly observed in people who are overweight and have high insulin levels (6,7), our study adds evidence of a beneficial effect of low-GI diets in healthy older women. Overall, weight loss did not reach a threshold considered to be clinically meaningful; however, moderate weight loss of 3% to 5% has been reported to improve health outcomes (18). Altogether, these results suggest that a diet with a low-GI and within the current RDA for protein intake (0.8 g/kg body weight) may have beneficial effects in reducing body weight and WC gains observed during the postmenopausal period.

On the other hand, neither the GI nor the protein content significantly influenced the participants’ glycolipid profile, which may be explained by the healthy status of our participants at baseline. In fact, our sample had good physical functioning and overall quality-of-life scores (11). Therefore, our results cannot be generalized to older women with comorbidities or physical limitations. Also, changes in glycolipid profile have been usually described in studies associated with energy restriction (19), and our intervention was intended to achieve energy balance. In addition, given the role of estrogens on glucose and lipid metabolism (20), the dietary intervention with a low-GI diet did not influence the glycolipid profile under the relative estrogen deprivation in healthy postmenopausal women.

Strengths of our study include its careful design, controlling for the variability in energy intake by prescribing a diet aimed to maintain energy balance, and measurements of insulin, glucose, total cholesterol, LDL-c, and HDL-c levels over 6 months of low-GI dietary intervention. One limitation of the present study is the analysis of secondary outcomes, as the trial was not originally designed to address these exploratory research questions. Other limitations include the relatively healthy status of participants and the absence of a control group receiving a high-GI diet.

In conclusion, protein intake exceeding the RDA did not lead to greater weight loss or WC reduction in a sample of healthy postmenopausal women following a low-GI diet for 6 months. Additionally, the glycolipid profile remained unchanged during the dietary intervention. Future RCTs will be important to confirm these results by comparing them to those in a control group receiving a high-GI diet for more than 6 months, especially in unselected late menopausal women.
